# Adolescent Loneliness and the Interaction between the Serotonin Transporter Gene (5-HTTLPR) and Parental Support: A Replication Study

**DOI:** 10.1371/journal.pone.0133430

**Published:** 2015-07-17

**Authors:** Annette W. M. Spithoven, Patricia Bijttebier, Wim Van Den Noortgate, Hilde Colpin, Karine Verschueren, Karla Van Leeuwen, Stephan Claes, Luc Goossens

**Affiliations:** 1 Department of School Psychology and Child and Adolescent Development, KU Leuven, Leuven, Belgium; 2 Department of Methodology of Educational Sciences, KU Leuven, Leuven, Belgium; 3 Department of Parenting and Special Education, KU Leuven, Leuven, Belgium; 4 Department of Neuroscience, KU Leuven, Leuven, Belgium; Radboud University, NETHERLANDS

## Abstract

Gene-by-environment interaction (GxEs) studies have gained popularity over the last decade, but the robustness of such observed interactions has been questioned. The current study contributes to this debate by replicating the only study on the interaction between the serotonin transporter gene (5-HTTLPR) and perceived parental support on adolescents’ peer-related loneliness. A total of 1,111 adolescents (51% boys) with an average age of 13.70 years (*SD* = 0.93) participated and three annual waves of data were collected. At baseline, adolescent-reported parental support and peer-related loneliness were assessed and genetic information was collected. Assessment of peer-related loneliness was repeated at Waves 2 and 3. Using a cohort-sequential design, a Latent Growth Curve Model was estimated. Overall, a slight increase of loneliness over time was found. However, the development of loneliness over time was found to be different for boys and girls: girls’ levels of loneliness increased over time, whereas boys’ levels of loneliness decreased. Parental support was inversely related to baseline levels of loneliness, but unrelated to change of loneliness over time. We were unable to replicate the main effect of 5-HTTLPR or the 5-HTTLPR x Support interaction effect. In the Discussion, we examine the implications of our non-replication.

## Introduction

The concept of Gene-by-Environment interactions (GxE) refers to the idea that the effect of the environment on the individual’s phenotype depends on this individual’s genotype [1]. Although GxE studies have become increasingly popular [2], the robustness of such interactions has been questioned as many replication attempts yielded non-significant results [3, 4]. No replication studies have been published on the GxE effects on loneliness [5]. The current study contributes to the discussion by investigating the replicability of findings concerning the interaction between a well-known polymorphism in the serotonin transporter gene (5-HTTLPR) and perceived parental support as it affects adolescents’ loneliness.

Loneliness refers to subjective feelings of social isolation or lack of connectedness [6]. It has been related to various clinically relevant problems and disorders [7]. As loneliness is assumed to increase during adolescence [7, 8], examining predictors of loneliness during this developmental stage is important. Factors associated with the development and maintenance of loneliness might be found in the environment as well as in the individual. Within the environment, parenting behaviors have an established relationship with loneliness [9]. More specifically, positive parenting behaviors, like social support, have been associated with lower levels of loneliness, whereas negative behaviors, like hostility or criticism, have been related with higher loneliness levels [10, 11].

Within the individual, attention has recently turned to genetic factors, as the heritability of loneliness is estimated to be between 37 and 55% [12–14]. However, these estimates do not give an indication where the genetic risk originates from. One way to discover the genetic origin is by studying whether variations of the same gene (i.e., different alleles) are associated with loneliness [5]. In the domain of internalizing problems, 5-HTTLPR is a promising candidate gene. 5-HTTLPR has most often been examined in a short and long allelic variant, of which the short allelic variant is seen as the vulnerability factor for internalizing problems [11, 15]. More specifically, the short allelic 5-HTTLPR variant has been related to an oversensitivity to environmental stress and environmental threats. Social isolation, because of the relevance of social contact to survival, might elicit a stress and/or threat-response [16]. Therefore, it might be expected that individuals with at least one short 5-HTTLPR allele have a stronger reaction to social isolation, and thus experience more severe feelings of loneliness [11].

In the current paper, we try to replicate the findings of van Roekel et al. [11] who examined 5-HTTLPR in relation to adolescents’ peer-related loneliness. They found a main effect of this polymorphism on the change of adolescent loneliness over time. The loneliness levels of adolescents carrying a short allele remained stable over time, whereas the loneliness level of adolescents carrying the long allele decreased. In addition to this genetic main effect of 5-HTTLPR on the course of loneliness, an interaction effect of the polymorphism with perceived parental support on the baseline level of adolescents’ loneliness was found. Adolescents who carried a short allele and who reported high levels of support showed a lower baseline level of loneliness than both adolescents with a short allele who experienced low levels of support and adolescents with long allele (irrespective of the perceived level of support) [11].

In addition to the genetic effects, van Roekel et al. [11] also examined gender effects. Cross-sectional evidence on gender differences in loneliness is inconsistent. Many studies did not find gender differences in loneliness [8, 17]. Some studies indicated that men report more loneliness than women [18, 19], whereas other studies indicated that women report more loneliness than men [20, 21]. In the study by van Roekel and colleagues [11] girls reported to have higher levels of loneliness over time than boys. As sex hormones might have a differential effect on gene expression [22], they examined whether the genetic effects on loneliness were moderated by gender and vice versa. However, they were not able to find a 5-HTTLPR x Gender interaction effect.

## Method

### Participants and Procedure

Data for the current study were taken from the STRATEGIES project (acronym for *Studying Transactions in Adolescence*: *Testing Genes in Interaction with Environments)*. For this project, secondary schools from Flanders, the Dutch-speaking part of Belgium, offering various educational tracks (i.e., general, technical, and vocational education) were invited to participate. Nine schools participated, from which 121 seventh, eighth, and ninth grade classes were selected. Active written consent from parents and adolescents was required for participation. A total of 1,116 adolescents (49.50%) agreed to participate. The response rate of the current study falls within the range of response rates of other survey studies [23]. At each measurement occasion, researchers visited the schools to assist the participating adolescents in completing the questionnaire in a 50-minute session. In case the adolescents were not able to complete the questionnaire in time, they were asked to do so at home and return the completed questionnaire within 2 weeks. Five participants had to be dropped because they filled out the questionnaire in an unreliable way. The STRATEGIES study was approved by the Institutional Review Board of the Faculty of Medicine at the University of Leuven.

The final analytic sample at the first year of data collection (*N* = 1,111; 51.00% boys) was on average 13.70 years old (*SD* = 0.93). Of the adolescents, 36.00% were in Grade 7 (*M*_age_ = 12.89, *SD*_*age*_ = 0.49), 37.40% were in Grade 8 (*M*_age_ = 13.85, *SD*_*age*_ = 0.48) and 26.60% were in Grade 9 (*M*_age_ = 14.90, *SD*_*age*_ = 0.50). The majority of the participants (91.00%) had European grandparents. The reported parental education level, with 32.20% of the parents being a high school graduate, 41.60% of the parents having a bachelor’s degree, and 13.40% of the parents having a master’s degree, is comparable to the Flemish population aged 25 to 64 [24].

The drop-out rate was low: 75.20% of the participants filled out the loneliness measure on all measurement occasions, 15.40% had a missing value on one measurement occasion, 8.90% had missing values on two measurement occasions, and 0.50% did not fill out the loneliness measure. The different grades did not differ in the pattern of missing values (χ^2^ (6) = 11.52, *p* = .074). For 41 of the adolescents (3.70%) no information on their genotype was available, due to the adolescent being absent during saliva collection (*n* = 7), dropping out of the study (*n* = 6), or a technical failure during genotyping (*n* = 28). Adolescents with and without genotypic data did not differ significantly (*p* > .05) in age, loneliness, or parental support at Wave 1. The distribution across gender did not differ significantly either (*p* = .649). The adolescents without genotype information were retained in the sample.

### Measures

#### Loneliness

At all three measurement occasions, the participants filled out the 12-item peer-related loneliness subscale of the Louvain Loneliness Scale for Children and Adolescents (LLCA; [25]). The same scale was also used in the study by van Roekel et al. [11] A sample item from this subscale is “I feel sad because I do not have friends”. The participants rated the items on a 4-point Likert scale, ranging from 1 (*never*) to 4 (*often*). For all individuals, a mean score was calculated, with higher scores indicating higher levels of peer-related loneliness. The Cronbach’s alphas for the three measurement occasions were .91, .90 and .91, respectively, indicating excellent internal consistency.

#### Perceived parental support

In the current study adolescents reported on perceived parental support at baseline. Van Roekel et al. [11] measured support with items concerning appreciation, love, and encouragement as well as items concerning criticism, humiliation, and aggression. In order to approximate the content of this measure as closely as possible, we measured support using the Responsivity subscale (7 items; e.g., “My parents give me a lot of care and attention”) of the Louvain Adolescent Perceived Parenting Scale (LAPPS; [26]) as well as the Hostility subscale (6 items; e.g., “My parents say mean things to me”) of the Verbal Hostility Scale [27]. For both subscales, the items were rated on a 5 point-Likert scale, ranging from 1 (*never)* to 5 (*always)* and the Hostility subscale items were reverse coded. For each participant, a total score based on the 13 items was calculated, with higher scores indicating higher parental support. Cronbach’s alpha for the support scale was .85, indicating good internal consistency.

#### Genotyping

We examined the functional polymorphism, consisting of a short and a long version, in the promoter region (5-HTTLPR) of the serotonin transporter gene. Saliva samples, from which the DNA was derived, were collected at the first measurement wave using Oragene DNA collection kits (DNA Genotek; Ontario Canada). For genotyping, a fragment analysis, preceded by a Polymerase Chain Reaction (PCR), was conducted. The amplification mixture for PCR contained 12.5 μl Master Mix (Promega), 0.5 μmol/L of forward primer (5’-CAACCTCCCAGCAACTCCCTGTA-3’), 0.5 μmol/L of reverse primer (5’-GAGGGACTGAGCTGGACAACCAC-3’), 50ng DNA and 1.5 μl water. The PCR cycling conditions were 5 minutes at 95°C, 35 cycles of 30 seconds at 95°C, of 30 seconds at 60°C and of 90 seconds at 72°C, followed by a final extension of 7 minutes at 72°C. For the fragment analysis 0.5 μl of the PCR product with 0.5 μl GeneScan 600 LIZ Size Standard v2.0 (Applied Biosystems) and 10 μl Hi-Di formamide was used. After a denaturation of 3 minutes at 95°C the analysis was conducted in an ABI 3730xl Genetic Analyzer (Applied Biosystems). The results were printed with GeneMarker software Version 1.91 [28]. The fragment analysis was conducted for both alleles of the gene separately. There were no deviations found from the Hardy-Weinberg equilibrium, which indicated that allele and genotype frequencies of our sample were similar to what would be expected in the population (*p* > .98). Heterozygote profiles (i.e., short-long) or homozygote profiles (i.e., short-short or long-long) were constructed for each participant. It is assumed that carrying at least one short allele is a vulnerability factor. Following van Roekel et al., the allelic profiles were dummy-coded so that 0 was used for the short-short and short-long allelic profile and 1 was used for the long-long allelic profile [11].

### Statistical Analyses

The STRATEGIES project used a cohort-sequential or accelerated longitudinal design, in which three cohorts (starting in Grades 7, 8, and 9, respectively) were followed over a three-year period. Cohort differences in loneliness were tested using multilevel analysis, with measurement occasions (Level 1) nested within students (Level 2). This test revealed that there was no main effect for cohort (*F* (2, 1331.37) = 1.68, *p* = .187) nor a Cohort x Time interaction effect (*F* (2, 1182.28) = 1.51, *p* = .221). Thus, the overlapping and adjacent segments of the three-year longitudinal data from the different cohorts could be linked in such a way that each cohort contributes to different, though partly overlapping, sections of the overall growth curve.

Assuming the GxE interaction explains between 1%, and 10% of the variance in loneliness, a study by Duncan and Keller (4) showed that a sample size between 500 and 1,000 participants would be sufficient to reach a power level of .80. However, this estimate was based on studies with a cross-sectional design, while we used a longitudinal design. Therefore, we conducted a Monte Carlo simulation in Mplus to estimate the power of our final model, which included all study variables. We based our expectations on the combined findings of the paternal and maternal models of the van Roekel et al. studies [11, 29]. These were the only studies on loneliness, measured support for the GxE interaction or had an longitudinal design. Our power analysis indicated that with a sample size of 1,100 participants the power was estimated to range between .11 and .31, except for three effects that had a power below .10. This indicates that our last and most complex model is underpowered. However, the power for the other, less complex models is expected to be higher.

Following van Roekels’ et al. [11] analysis, a Latent Growth curve model (LGCM) was estimated using structural equation modeling in Mplus [30]. To link the adjacent segments of data from different cohorts, we used the Mplus data cohort command. As the dependent variables had non-normal distributions, maximum likelihood with robust standard errors (MLR) was chosen as estimator in combination with Full Information Maximum Likelihood (FIML) to handle missing data. First, a basic growth model with only intercept (i.e., baseline level of loneliness) and slope (i.e., change in loneliness) was estimated. Second, the gene variable (5-HTTLPR) was introduced into the model, in order to examine its relationship with the onset and development of loneliness. Third, gender and the 5-HTTLPR x Gender interaction was examined in relationship to both the baseline of loneliness and its change over time. Finally, the relation between perceived parental support and loneliness as well as the relation between the 5-HTTLPR x Support interaction and loneliness were examined. Model fit was assessed by using the χ^2^, CFI, (with a value of .95 regarded as the cut-off for a good fitting model) and RMSEA (with a cut-off value of .05) [31]. Data and models can be found on https://osf.io/b7tf9.

## Results

### Descriptive Statistics

Out of the 1,111 participants, 202 adolescents (18.20%) were homozygous for the short allele, 520 adolescents (46.80%) were heterozygous, and 348 adolescents (31.30%) were homozygous for the long allele. Dummy-coded allelic profiles were used in the model. It should be noted that these descriptive statistics were comparable to the ones reported in the van Roekel et al. study [11].

### Model Findings

The basic model without predictors had a good fit, χ^2^(7) = 13.15, *p* = .069, *RMSEA* = .03 and *CFI* = .99. The model indicated that adolescents’ baseline loneliness score was on average 1.53 (*SE* = .02, *p* < .001), with individual adolescents differing significantly around this mean score (σ^2^ = .18, *SE* = .01, *p* < .001). On average, the adolescents showed a slight linear increase of loneliness over time (*M*_*s*_ = .02, *SE* = .01, *p* = .024), with individual adolescents having significant variation around this trajectory (σ^2^ = .02, *SE* = .03, *p* = .003).

In the second model, the 5-HTTLPR polymorphism was included as predictor of the baseline level of loneliness and its change (χ^2^(10) = 14.93, *p* = .135, *RMSEA* = .02 and *CFI* = .99). The 5-HTTLPR polymorphism was not significantly related to the baseline level of loneliness (B = -.02, *SE* = .04, p = .583) nor to change of loneliness over time (*B* = .02, *SE* = .02, *p* = .346). Consistent with this pattern of findings, the explained variability of the baseline loneliness level and change of loneliness over time was low. For both model parameters it was less than 1%.

**Fig 1:**
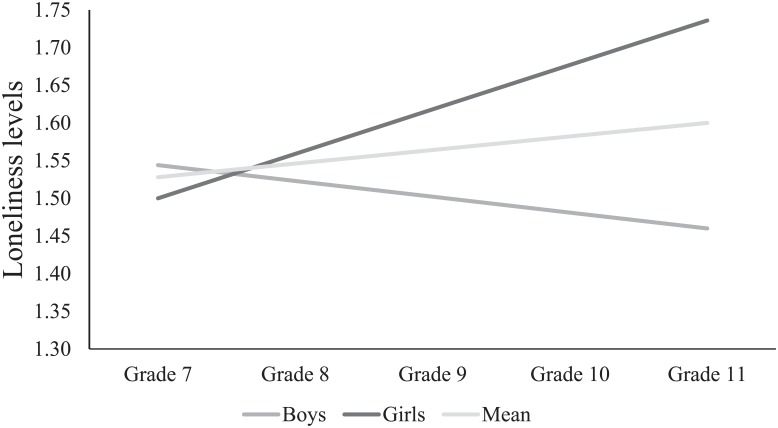
Loneliness scores over time for boys and girls separately. On average, loneliness levels increased over time. When separated for gender, only girls’ levels of loneliness increased over time, whereas boys’ levels of loneliness decreased.

In the third model, gender and the 5-HTTLPR x Gender interaction were introduced (χ^2^(16) = 17.90, *p* = .330, *RMSEA* = .01 and *CFI* = .99). Gender was not related to the baseline level of loneliness (*B* = -.04, *SE* = .05, *p* = .453). The relation of gender with the change of loneliness was significant (*B* = .08, *SE* = .02, *p* < .001). As seen in Fig 1, girls’ levels of loneliness significantly increased over time (*p* < .001), whereas boys’ levels of loneliness decreased (*p* = .049). The 5-HTTLPR x Gender interaction was not significantly related to the baseline level of loneliness (B = -.02, *SE* = .09, *p* = .847) or change of loneliness over time (*B* = -.03, *SE* = .04, *p* = .342). Whereas the explained variability of the baseline level of loneliness remained below 1%, the explained variability of the change in loneliness was 11.70%.

Finally, support and the 5-HTTLPR x Support interaction were introduced into the model (χ^2^(22) = 27.02, *p* = .210, *RMSEA* = .02 and *CFI* = .99). A main effect of parental support on the baseline level of loneliness was found (*B* = -.23, *SE* = .05, *p* < .001). Adolescents who perceived their parents as more supportive reported a lower baseline level of loneliness than adolescents who perceived their parents as less supportive. However, support was not related to the change of loneliness over time (*B* = .02, *SE* = .02, *p* = .179). The 5-HTTLPR x Support interaction was not significantly related to the baseline level of loneliness (*B* = .07, *SE* = .07, *p* = .352) nor to the change of loneliness over time (*B* = .01, *SE* = .03, *p* = .797). The explained variability of the baseline level of loneliness was 9.80%, whereas the explained variability of the change in loneliness was 14.50%.

## Discussion

In the current paper, we tried to replicate the study of van Roekel and colleagues [11] on the effect of 5-HTTLPR and the interaction effect of 5-HTTLPR and perceived parental support on adolescent loneliness over time. Although the overall design of the current study was highly comparable to the study by van Roekel et al. [11], we were unable to replicate their main or interaction effect for the serotonin transporter gene. In line with the van Roekel et al. [11] study, parental support was related to lower levels of loneliness at baseline and girls had higher loneliness levels over time than boys.

Parental support seems to be a buffer against adolescent peer-related loneliness at baseline. This finding seems to be consistent with the broader literature, in which parental support is negatively related to internalizing problems in general [32]. The relation between parental support and adolescent loneliness can be explained in different ways. First, parents might affect their children’s reaction to peers by modeling, evoking, and reinforcing certain problem-solving strategies and social behaviors. These problem-solving strategies and social behaviors, in turn, might affect the children’s relationships with peers and feelings of peer-related loneliness [33]. Second, children develop perceptions and expectations of social interactions in reaction to their parents’ parenting behaviors. Their representations of the parent-child relationship are generalized to the peer context [9]. These generalized representations and expectations might influence the child’s problem solving strategies as well as their behavior towards peers, which both influence peer evaluations and responses [33].

Neither the main effect of 5-HTTLPR on loneliness nor the 5-HTTLPR x Support interaction on loneliness were replicated in the current study. However, our findings are in accordance with two meta-analyses that did not find evidence for an interaction effect of 5-HTTLPR and stressful life events on depression either [34, 35]. Another meta-analysis [36] suggested that the moderation effects of 5-HTTLPR may be more pronounced in childhood. It might be that the effect of 5-HTTLPR is less clearly expressed in adolescence. Also, the influence of other environmental factors may have confounded the 5-HTTLPR and parental support interaction [37]. In addition, the effects of 5-HTTLPR might not be revealed by using a short and long allelic dichotomy. Growing evidence suggest that using a dichotomy for 5-HTTLPR is too simplistic, because its functionality seems to be determined by various other genetic factors [15].

Previous research already showed that it is hard to find robust GxE effects using polymorphisms in a single gene [4]. This is not completely explained by studies being low in power, as well-powered studies might also yield substantially varying effect estimates. Low power just increases the range of effect estimates one might find [38]. Therefore, when multiple replication studies are conducted, a meta-analysis or integrative data analysis should be used to combine the results of various studies to find the true effect of interest as well as its appropriate magnitude [39]. In addition, an inverse relation between statistical power and the support for GxE interactions has been reported [40]. Considering all this, future GxE studies should pay close attention to statistical power. Alternative ways to increase power, other than increasing sample size, should be considered. As the effects of single genes are assumed to be small [2], genetic effect might be increased by taking multiple genes into account. One way to do so is by calculating polygenic risk scores [40, 41].

Cross-sectional studies on gender differences in loneliness have led to contrasting results, with most studies finding no gender differences [17, 19, 20]. Using baseline loneliness data, which are roughly similar to comparisons at a single point in time, we did not find gender differences. However, we were able to replicate van Roekel and colleagues’ [11] finding that girls scored higher on loneliness over time than boys. It would be interesting for future studies to take a closer look at gender differences in loneliness over time.

The current replication study has some limitations that should be mentioned. First, all measures were based on self-report, which causes shared method variance. However, given that loneliness is a subjective and internal experience, self-report has been put forward as the most adequate assessment method [42]. In addition, adolescents’ report on parenting behavior might be preferred as well. Adolescents’ perception of parenting behavior might have a stronger association with adolescents’ adjustment than parents’ actual parenting behavior or the parents’ perception of it [43, 44]. Second, for practical reasons, the adolescents in our study reported on parental support in a general sense instead of reporting on maternal and paternal support separately. The decision to assess perceived support for both parents combined was based on a study indicating a high correlation between maternal and paternal parenting behaviors [45]. Nevertheless, van Roekel et al. [11] did find differential effects for maternal and paternal support, which we were unable to examine. The third limitation concerns environmental factors. In line with the study of van Roekel et al. [11], the environment factor, support, was only measured once in the current study. However, the effect of the environment factor might be different from one time point to another. It is possible that, over time, the sensitivity of the individual for the environmental factor changes or that the environment factor itself changes. Therefore, it would be more developmentally sensitive to take parental support over time into account as well [46]. In addition to the measurement of support, it should be noted that other variables might have an effect on adolescents’ loneliness, such as mental health problems, social-economical context, relations with peers, and other parenting behaviors. Future studies might also include both positive and negative environmental factors, because individuals are most likely genetically sensitive to both positive and negative environmental aspects [37]. Finally, given the correlational design of the current study, no conclusions about causality can be drawn.

## Conclusion

The current study tried to replicate the interaction effect of 5-HTTLPR and perceived parental support on adolescents’ peer-related loneliness [11]. Although we did replicate the main effects for parental support and gender, we were unable to replicate the 5-HTTLPR main or interaction effects. It seems hard to find robust GxE effects using polymorphisms in a single gene. Future studies might consider alternatives for the single gene-single location approach as a way to increase statistical power.
